# Surface-Metric Strain Estimation from Three-Dimensional Digital Image Correlation for CTE Measurement of Warping Electronic-Packaging Films

**DOI:** 10.3390/ma19214576

**Published:** 2026-10-29

**Authors:** Shen Liu, Yuanqiang Chen, Chaoqian Huang, Shuai Wang, Fang Yuan, Kang Wei, Cong Liu, Zhuoyi Yin

**Affiliations:** 1 School of Physics, Nanjing University of Science and Technology, Nanjing 210094, Chinacyq704010572@njust.edu.cn (Y.C.);; 2 School of Civil Engineering, Suzhou University of Science and Technology, Suzhou 215009, China; yuanf@usts.edu.cn; 3 School of Civil Engineering, Southeast University, Nanjing 210096, China

**Keywords:** three-dimensional digital image correlation, coefficient of thermal expansion, electronic packaging, thin film, warpage, surface metric, Green–Lagrange strain

## Abstract

Thin electronic-packaging films can develop substantial warpage during heating. Three-dimensional digital image correlation (3D-DIC) reconstructs the full displacement field, yet displacement-plane fitting still introduces curvature-dependent projection errors into the calculated strain. The proposed estimator differentiates matched reference and heated surfaces in common material coordinates and compares their first fundamental forms. Directional Green–Lagrange strains are then averaged over a fixed region of interest before temperature regression. Both global-coordinate and local-plane DPF varied with curvature in the cylindrical benchmarks. The surface-metric CTE remained invariant at the reporting resolution across the tested bending directions and regions of interest. Coordinate-noise simulations quantified precision after field averaging and regression. In the reported copper-foil sequence, the surface metric gave an RMSE of 0.170 $\mathrm{ppm}\;K^{-1}$ relative to the NIST copper reference, which was lower than either DPF baseline. The method measures directional CTE during smooth developable warpage without prescribing its shape, curvature, or bending direction.

## 1. Introduction

Electronic packages combine silicon, copper, polyimide (PI), polymer dielectrics, epoxy molding compounds, and metallized redistribution layers within a mechanically coupled stack [1,2,3,4]. Their coefficients of thermal expansion (CTEs), elastic moduli, cure histories, and viscoelastic responses differ substantially. Heating during cure, reflow, qualification, or service creates incompatible thermal eigenstrains. The resulting membrane forces and bending moments drive chip and package warpage, registration error, interfacial stress, and the fatigue of interconnects [1,5,6]. Accurate CTE recovery must account for surface shape because the desired in-plane thermal strain can accompany a much larger out-of-plane displacement.

Digital image correlation (DIC) provides non-contact, full-field measurements, with speckle targets and coating methods supporting surface tracking [7,8]. Camera configuration affects reconstruction precision [9]. Thermal applications include heat-wave compensation and measurements on heated steel plates [10,11]. Established film measurements correlate a reference image with heated images, calculate local strain in a spatial window, average each normal-strain component over a fixed region of interest (ROI), and obtain directional CTE from the corresponding strain–temperature slope [12,13,14]. Contrast enhancement and high-throughput implementations have extended the range of measurable polymers and thin-film surfaces [15,16]. Fixed-ROI averaging attenuates approximately zero-mean, high-frequency strain fluctuations while retaining directional information.

A CTE difference of 1 $\mathrm{ppm}\;K^{-1}$ produces only 10 με over a 10 K interval. Resolving this increment in one local-strain estimate is difficult in thermal stereo-DIC because spatial differentiation amplifies coordinate noise and the heated optical path adds image fluctuations. Established CTE workflows, therefore, average strain over a large field before fitting the temperature slope [12,13,14]. This treatment is effective for thick, homogeneous coupons with modest shape changes. Thin foils can release residual stress through much larger curvature changes. Field averaging reduces random fluctuations but retains the systematic contribution of a planar projection. The present method keeps the fixed ROI and temperature regression and replaces the projected local strain with a surface-metric strain.

Stereo DIC reconstructs matched surface points in three dimensions and can report in-plane displacement and warpage in the same experiment [17,18,19]. This capability has been applied to package substrates, ball-grid-array structures, and thermally loaded assemblies [20,21,22]. Strain accuracy then depends on the coordinates supplied to the derivative calculation. The classical displacement-plane fit (DPF) is reliable for locally affine displacement fields. Laboratory X/Y coordinates introduce direct foreshortening, while an independently fitted local plane represents a curved neighborhood by one average plane. Both projections vary with curvature during isometric bending.

**Figure 1:**
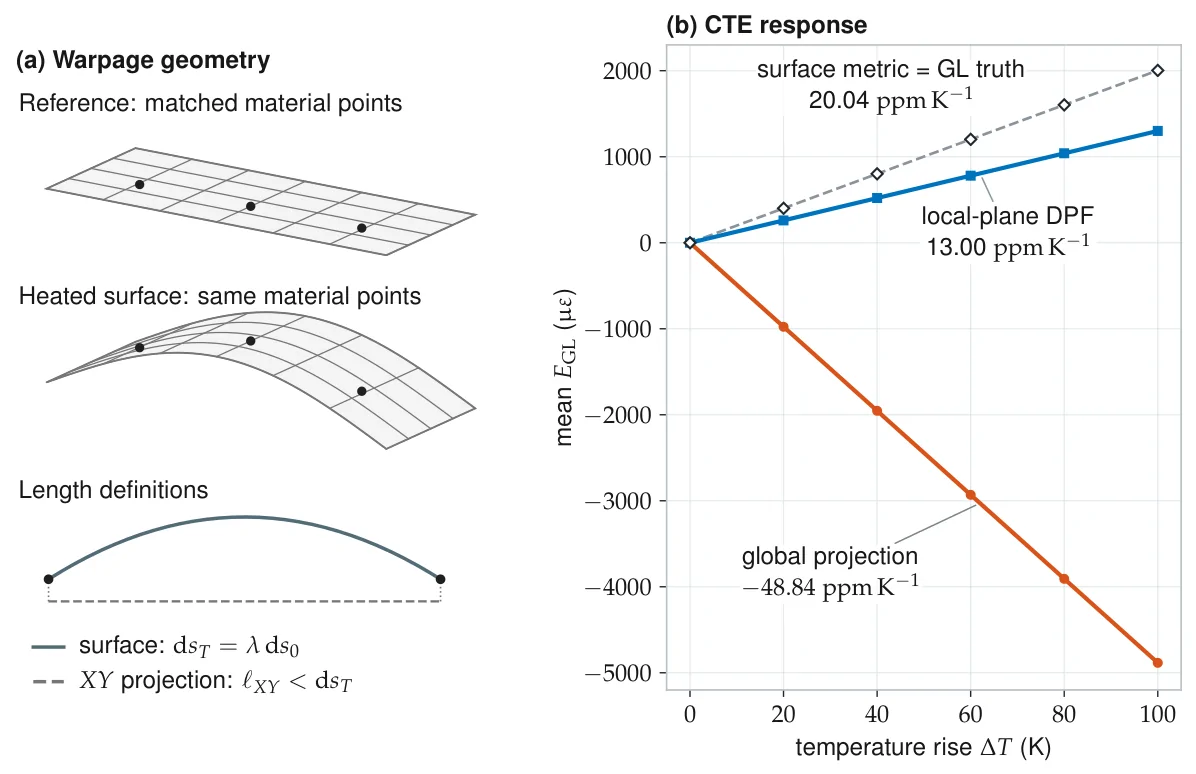
Warpage-induced CTE bias. (**a**) Matched surfaces and material distances (schematic, not to scale). (**b**) Fitted mean direct-Green–Lagrange strain trends.

Figure 1 illustrates how this geometric choice enters CTE measurement. The reference and heated surfaces contain the same matched material points. The surface metric evaluates their local material distances, while the two DPF calculations use planar representations of the curved neighborhood.

Planar DIC studies established CTE extraction from mean thermal strain [12,13,14], and packaging studies measured warpage and in-plane response simultaneously [20,21,22,23]. Curved-surface strain has been represented in several ways. Local tangent-frame calculations express neighborhood deformation relative to the measured surface normal and are sensitive to normal variability [24]. Finite-element stereo-DIC represents shape and displacement on a global mesh and can incorporate mechanical regularization [25]. Isogeometric and surface-parameterized formulations describe displacement and strain directly on complex surface geometries [26,27,28]. Thermal applications include tubular steel specimens and composite laminates [29,30]. Graciani et al. [31] determined directional in-plane CTEs of carbon-fibre/epoxy angle brackets from mean thermal strains. They also used out-of-plane displacements to measure angular distortion and estimate through-the-thickness CTE. The present study evaluates how the local-strain observable affects CTE recovery as film curvature changes. Matched reference/current first fundamental forms are tested with a developable benchmark that isolates curvature-dependent DPF bias, while retaining the fixed-ROI average and temperature regression.

Thermal imaging adds coordinate uncertainty through image noise, speckle gradients, subset overlap, interpolation, and the correlation algorithm [32]. Camera self-heating can change calibration parameters [33,34], while temperature gradients can distort both stereo views [10,35,36]. Temporal or spacetime processing suppresses part of this optical fluctuation [37]. Optical effects determine coordinate precision. The strain formulation determines the curvature-dependent contribution to the recovered CTE.

This study compares the first fundamental forms of matched reference and heated surface neighborhoods. A cylindrical benchmark varies curvature while preserving the prescribed in-surface stretch, so any change in the recovered CTE measures projection error. Smooth nonuniform and non-developable fields extend the evaluation to spatially varying surface geometry.

The estimator uses the reference coordinates, matched displacement increments, material-grid topology, validity mask, and ROI mask available after stereo reconstruction. Film thickness, warpage shape, curvature, and bending direction do not enter the calculation. We compare global-coordinate DPF, local-plane DPF, and the surface metric across curvature, direction, and ROI. Coordinate-noise simulations and copper-foil experiments evaluate the resulting CTE workflow.

## 2. Materials and Methods

### 2.1. Three-Dimensional DIC Acquisition and Reconstructed Fields

A calibrated stereo pair observes a random-speckled film in a reference state $T_{0}$ and at heated states $T_{k}$. Subset correspondence in the two camera views and stereo triangulation provide the reference coordinate of material-grid point (i,j),

(1) $$X_{ij}={\left[\begin{array}{ccc} X_{ij} & Y_{ij} & Z_{ij} \end{array}\right]}^{T},$$

and its matched displacement

(2) $$D_{ij}\left(T_{k}\right)={\left[\begin{array}{ccc} D_{X,ij} & D_{Y,ij} & D_{Z,ij} \end{array}\right]}^{T}.$$

**Figure 2:**
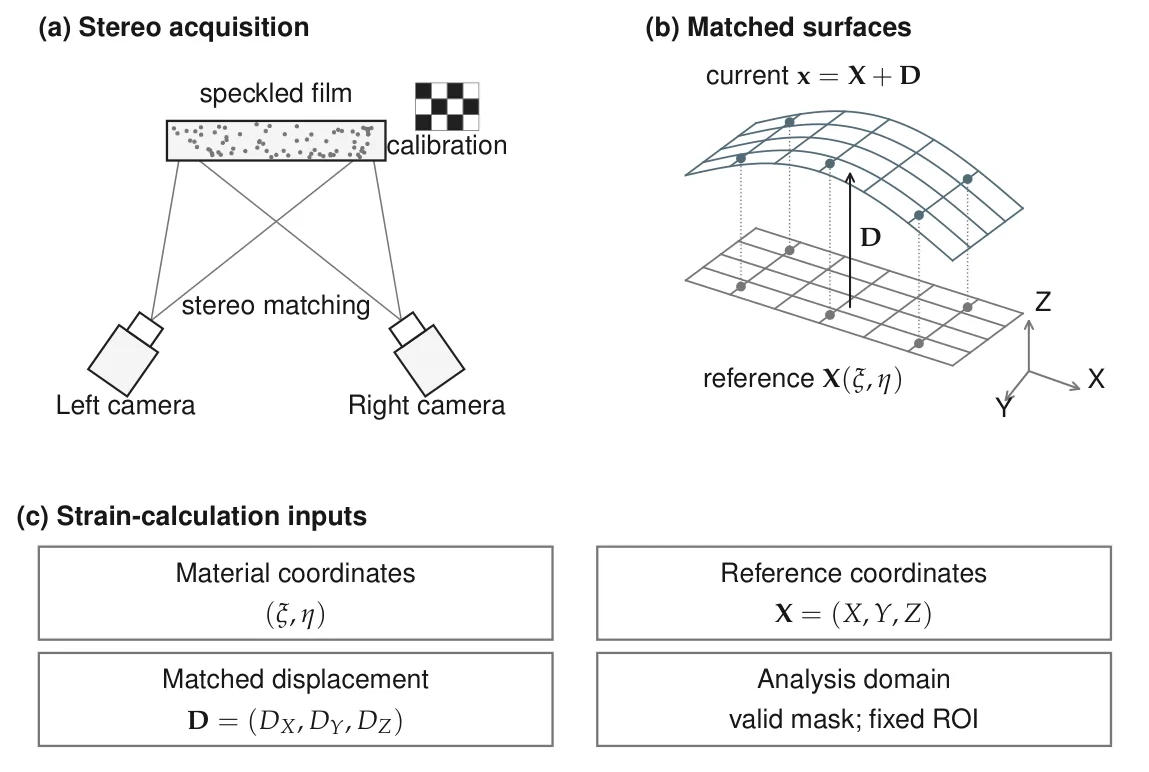
3D-DIC measurement (schematic, not to scale). (**a**) Stereo acquisition. (**b**) Matched reference and heated surfaces. (**c**) Strain-calculation inputs.

The current coordinate is $x_{ij}\left(T_{k}\right)=X_{ij}+D_{ij}\left(T_{k}\right)$. Row and column connectivity defines a local, dimensionless material parameterization (ξ,η) derived from the discrete topology. A validity mask excludes failed correlations, and one fixed ROI is used at every temperature. Figure 2 illustrates the acquisition and the matched reference/current surfaces. Section 4 reports the copper-foil heating, stereo hardware, calibration, and correlation parameters. The surface-metric calculation operates on the coordinate fields produced by camera calibration, stereo matching, and triangulation.

The calculation returns the in-surface Lagrangian strain of the observed material surface. Local derivatives are taken with respect to the common material grid, using the matched three-dimensional reference and current coordinates. Temperature regression follows the separate fixed-ROI averages of $E_{xx}$ and $E_{yy}$ for each frame.

### 2.2. CTE Estimand and Strain Convention

Let *x* and *y* denote specified material directions in the reference tangent plane. The analysis treats these directions separately and, therefore, accommodates anisotropic in-plane expansion. For a∈{x,y}, the directional stretch $\lambda_{a}$ and direct Green–Lagrange strain component $E_{aa}$ satisfy

(3) $$E_{aa}\left(T\right)=\frac{1}{2}\left[\lambda_{a}^{2}\left(T\right)-1\right],\;\lambda_{a}\left(T\right)=\sqrt{1+2E_{aa}\left(T\right)}.$$

For a reference state at $T_{0}$, the exact mean directional linear CTE over $\Delta T=T-T_{0}$ is

(4) $${\bar{\alpha }}_{a}\left(T_{0},T\right)=\frac{\sqrt{1+2E_{aa}\left(T\right)}-1}{\Delta T}=\frac{E_{aa}\left(T\right)}{\Delta T}+O\;\left(\frac{E_{aa}^{2}}{\Delta T}\right).$$

Thus, at the thermal strains considered here, the slope of direct Green–Lagrange strain is numerically equivalent to the conventional directional CTE. For example, at 20 $\mathrm{ppm}\;K^{-1}$ over a 10 K interval, the difference is approximately 0.002 $\mathrm{ppm}\;K^{-1}$. For each temperature frame, let $V_{k}$ denote the valid points within the fixed ROI. Each normal component is averaged independently,

(5) $${\bar{E}}_{aa}\left(T_{k}\right)=\frac{1}{N_{k}}\sum_{\left(i,j\right)\in V_{k}}E_{aa,ij}\left(T_{k}\right),\;a\in \left\{x,y\right\},\;N_{k}=\left|V_{k}\right|.$$

The two directional estimates are

(6) $${\hat{\alpha }}_{x}≃\mathrm{slope}\;\left[{\bar{E}}_{xx}\left(T_{k}\right)\right],\;{\hat{\alpha }}_{y}≃\mathrm{slope}\;\left[{\bar{E}}_{yy}\left(T_{k}\right)\right],$$

using one equal statistical weight per frame. The shear component $E_{xy}$ is retained as a deformation diagnostic. For the isotropic numerical benchmarks, scalar plots and Tables, we use ${\hat{\alpha }}_{\mathrm{avg}}=\left({\hat{\alpha }}_{x}+{\hat{\alpha }}_{y}\right)/2$. Experimental analyses retain the separate directional estimates.

The estimand is the temperature slope of the measured in-surface stretch under the imposed test-boundary condition. For a freely expanding film, ${\hat{\alpha }}_{x}$ and ${\hat{\alpha }}_{y}$ estimate the directional material CTEs under the small-strain direct-Green–Lagrange convention. For a bonded or mechanically loaded packaging layer, the same calculation reports its effective directional thermomechanical expansion in the assembled state. The metric calculation and its removal of projection artifacts are unchanged; the specimen configuration determines the physical interpretation of the slope.

### 2.3. Geometric Origin of Warpage Bias

Consider a smooth surface patch parameterized by material coordinates. Its reference and current embeddings are X(ξ,η) and x(ξ,η). A laboratory-plane calculation applies the projector

(7) $$P_{XY}=\left[\begin{array}{ccc} 1 & 0 & 0\\ 0 & 1 & 0 \end{array}\right]$$

to the three-dimensional tangent or displacement field. If a material line with stretch λ rotates by θ out of the laboratory plane, its projected direct-Green–Lagrange strain becomes

(8) $$E_{\mathrm{proj}}=\frac{1}{2}\left(\lambda^{2}\cos^{2}\theta -1\right).$$

The $\cos^{2}\theta$ term is a projection artifact. It varies with warpage amplitude, direction, and ROI, even when the material stretch is fixed.

A local plane fitted independently in the reference and current configurations represents each curved neighborhood by its average plane. For a smooth patch with $|\kappa L_{w}|\ll 1$, a local expansion gives a finite-window projection error of order $O(\kappa^{2}L_{w}^{2})$, where $L_{w}$ is the physical window span. The controlled benchmark below evaluates this curvature dependence directly.

### 2.4. Comparison of Local-Strain Estimators

The comparison uses two explicitly defined planar-window implementations of displacement fitting. Global-coordinate DPF fits only the laboratory $D_{X},D_{Y}$ components over the regular reference material grid, which coincides with reference X/Y in the flat benchmark; $D_{Z}$ does not enter its gradient. Direct Green–Lagrange strain is calculated from this two-dimensional fitted gradient, retaining the foreshortening term in Equation (8). Local-plane DPF fits the reference and current planes independently over the same square neighborhood, transforms matched three-dimensional points into the respective plane frames, and fits all three displacement components against the reference-plane coordinates. Direct Green–Lagrange strain follows from this affine gradient. In the deterministic benchmark, global-coordinate DPF uses a fixed 7×7 neighborhood, while local-plane DPF and the surface metric use fixed 9×9 neighborhoods. These supports are unchanged with curvature, direction, ROI, and temperature. For every estimator, the two normal strain components are averaged over the same ROI and regressed against temperature separately. Table 1 compares the geometric quantity calculated by each estimator.

**Figure 3:**
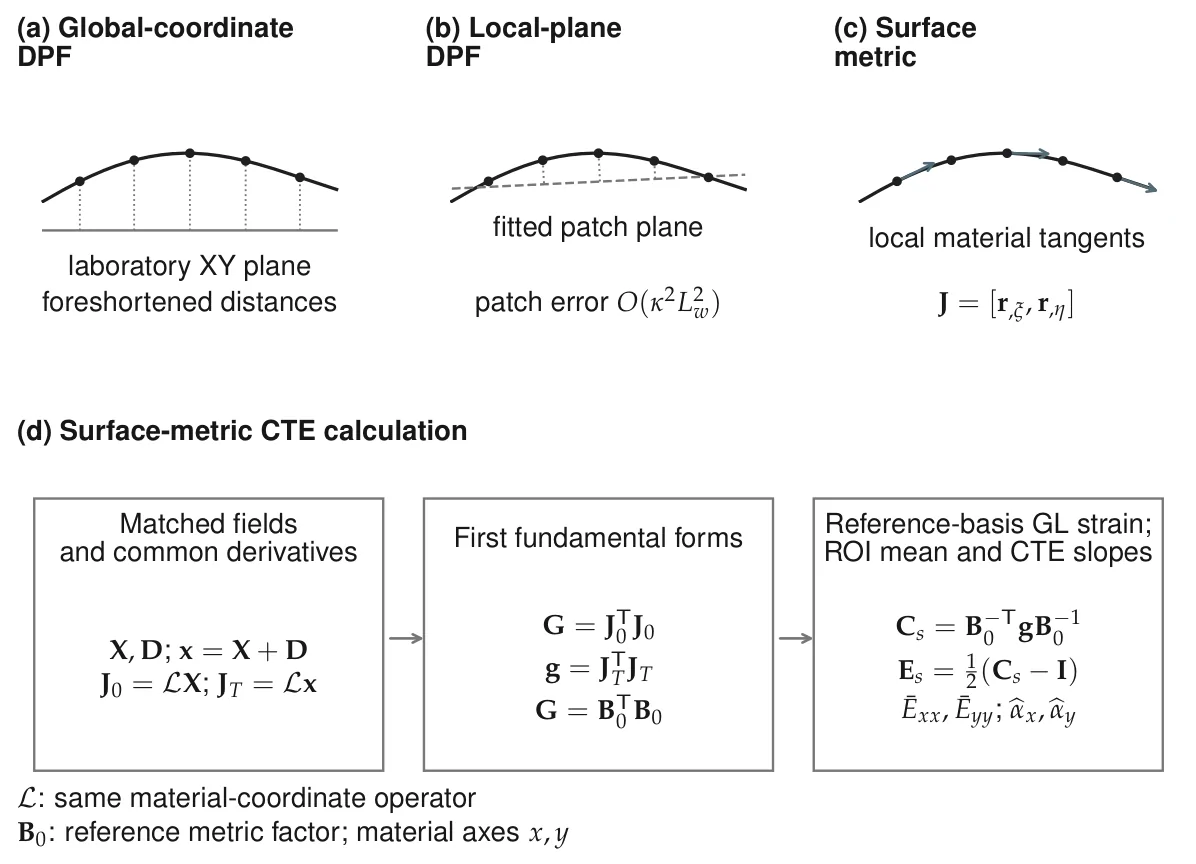
Local-strain estimators. Schematic sections (not to scale) show (**a**) global-coordinate DPF, (**b**) local-plane DPF, and (**c**) surface tangents. (**d**) Surface-metric strain and directional CTE calculation.

Figure 3 shows the global projection, the local fitted plane, and the surface-metric calculation using the same matched three-dimensional fields.

### 2.5. Surface-Metric Green–Lagrange Estimator

At each valid material point, the same fixed 9×9 material-grid neighborhood is used in the reference and current configurations. With centered and scaled coordinates $\left(\tilde{\xi },\tilde{\eta }\right)$, each Cartesian component $r_{q}$ of X or x is represented by the complete fifth-order polynomial

(9) $$r_{q}\left(\tilde{\xi },\tilde{\eta }\right)=\sum_{p+m\leq 5}a_{pm}^{\left(q\right)}{\tilde{\xi }}^{p}{\tilde{\eta }}^{m}.$$

The 81 samples overdetermine the 21 coefficients. Centering and scaling improve conditioning, and the symmetric support provides the two tangent derivatives at its center. The polynomial order and neighborhood remain fixed for every point, temperature, geometry, and estimator evaluation; Section 3.5 reports their numerical sensitivity. Differentiation at the center gives

(10) $$J_{0}=\left[\begin{array}{cc} X_{,\xi } & X_{,\eta } \end{array}\right],\;J_{T}=\left[\begin{array}{cc} x_{,\xi } & x_{,\eta } \end{array}\right].$$

Using the same linear derivative operator in both states holds the material basis and spatial support fixed during the metric comparison.

The reference and current first fundamental forms are

(11) $$G=J_{0}^{T}J_{0},\;g=J_{T}^{T}J_{T}.$$

Let $G=B_{0}^{T}B_{0}$ be a Cholesky factorization. The right Cauchy–Green tensor expressed in the reference orthonormal tangent basis is

(12) $$C_{s}=B_{0}^{-T}gB_{0}^{-1},\;E_{s}=\frac{1}{2}\left(C_{s}-I\right).$$

The reference tangent axes are oriented with the selected material *x* and *y* directions. Consequently, $E_{xx}={\left(E_{s}\right)}_{11}$ and $E_{yy}={\left(E_{s}\right)}_{22}$ enter the two independent CTE regressions in Equation (6). Equations (11) and (12) are objective under three-dimensional rigid motion and independent of laboratory orientation. Smooth isometric bending leaves the exact surface metric unchanged. Section 3 quantifies the discretization error of the fitted estimator. Changes in g directly produce the corresponding in-surface strain of the reconstructed surface.

Coordinate noise affects the estimated tangents and is evaluated separately in the numerical sensitivity study.

## 3. Numerical Simulations and Results

### 3.1. Coordinate Fields, Prescribed Metric, and Evaluation Measures

Every numerical case supplies the fields available after 3D-DIC reconstruction: a reference surface $X_{ij}$, matched displacements $D_{ij}$, row–column material topology, validity and ROI masks, and a temperature label for each frame. Isotropic thermal expansion, $\alpha_{x}=\alpha_{y}$, is prescribed in material coordinates before the surface is embedded in its warped shape. Each estimator receives the resulting coordinate fields and masks. Scalar results use ${\hat{\alpha }}_{\mathrm{avg}}$ because the benchmark is isotropic, while both directional estimates remain available.

The principal curvature–direction–ROI sweep uses a $20\times 20\;{\mathrm{mm}}^{2}$ surface sampled on a 101×101 grid (0.2 mm grid spacing) over 25–125 °C in 10 K steps. The 9×9 surface-metric neighborhood, therefore, spans 1.6 mm in this benchmark. The prescribed logarithmic CTE is $\alpha_{x}=\alpha_{y}=20.000$ $\mathrm{ppm}\;K^{-1}$, for which both direct-Green–Lagrange slopes over this finite interval are 20.040 $\mathrm{ppm}\;K^{-1}$. Deterministic cases are evaluated by the shift in ${\hat{\alpha }}_{\mathrm{avg}}$ from the corresponding flat baseline. Here, direction refers to the cylindrical bending orientation. In the noise study, one complete coordinate-field sequence is the statistical unit used to calculate CTE bias and RMSE.

**Figure 4:**
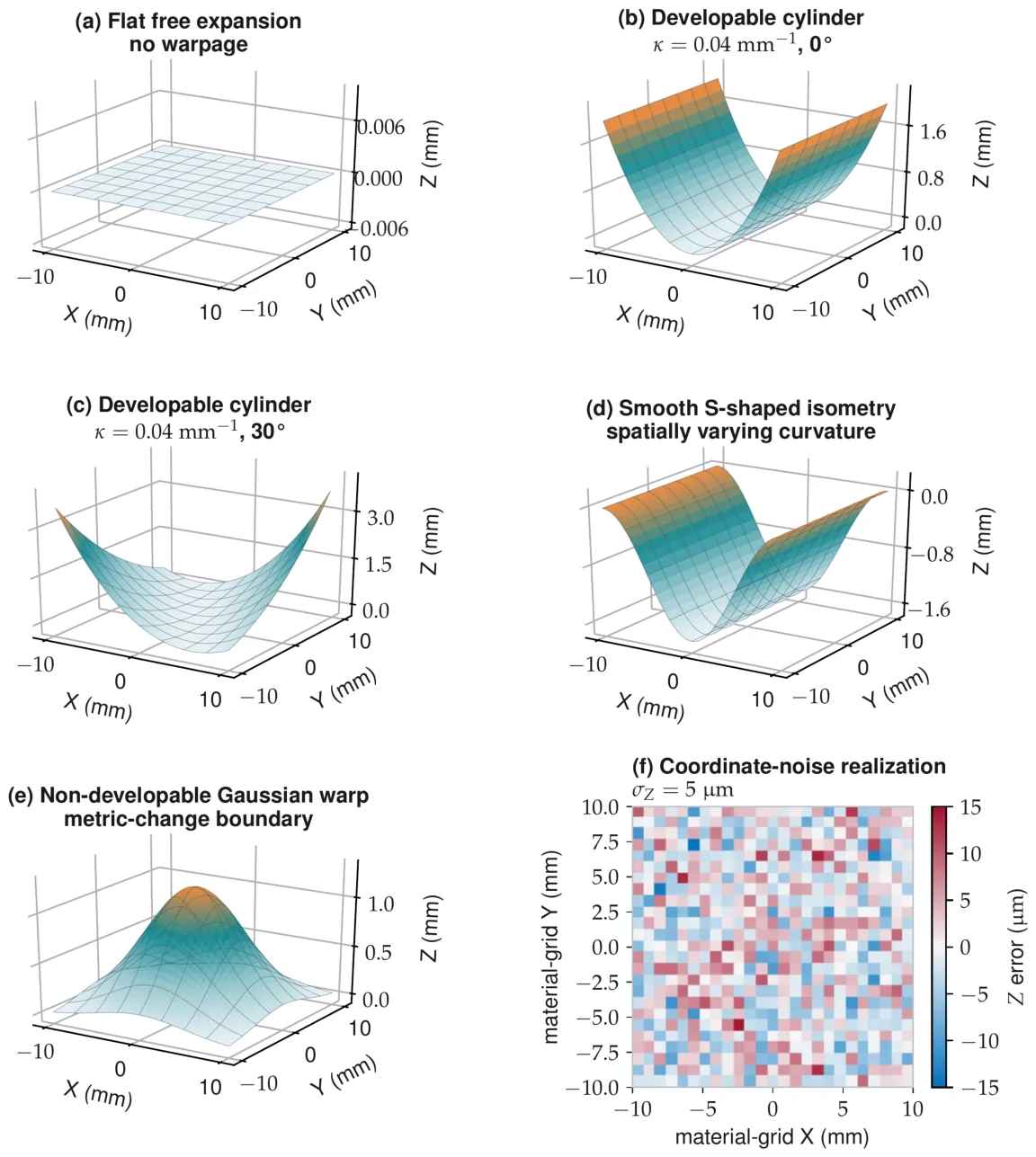
Simulated warpage fields. (**a**) Flat expansion. (**b**,**c**) Cylindrical bending. (**d**) Smooth S-shaped isometry. (**e**) Gaussian warpage. (**f**) Illustrative coordinate-noise realization. Colors in (**a**–**e**) indicate relative surface height within each panel; the *Z* axes give the numerical heights. In (**f**), color indicates *Z*-coordinate error in μm.

Figure 4 presents the field families used in the evaluation. The flat surface establishes the strain convention. Cylinders at two orientations test exactly developable bending, and the smooth S-shaped surface introduces spatially varying developable curvature. The Gaussian warp supplies a prescribed non-developable case with a changed first fundamental form. The final panel illustrates a realization of the imposed *Z*-coordinate noise.

### 3.2. Flat-Film Baseline and Strain Convention

The flat baseline contains uniform free expansion and no out-of-plane displacement. Global-coordinate DPF, local-plane DPF, and surface-metric strain act on the same planar distance field and recover the prescribed direct-Green–Lagrange slope of 20.040 $\mathrm{ppm}\;K^{-1}$. Its 0.040 $\mathrm{ppm}\;K^{-1}$ offset from the logarithmic input reflects the finite-strain convention in Equation (3). Unit tests confirm zero surface-metric strain under three-dimensional rigid translation and rotation. Warpage effects are reported as the change from each estimator’s flat result.

### 3.3. CTE Response Under Developable Warpage

**Figure 5:**
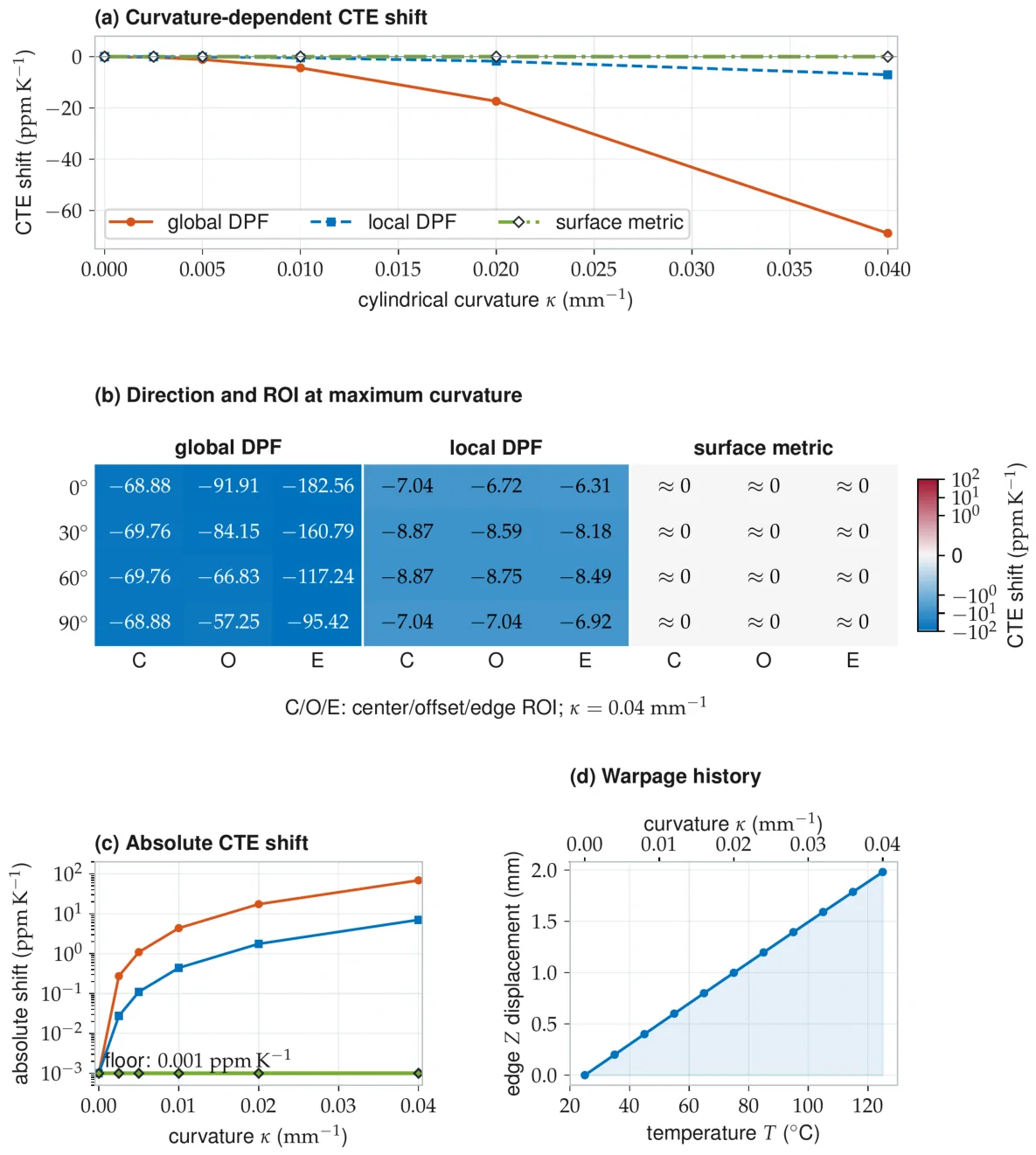
Deterministic warpage response. (**a**) Curvature dependence. (**b**) Direction and ROI dependence. (**c**) Absolute CTE shift on a logarithmic scale. (**d**) Prescribed curvature and representative edge displacement versus temperature. Values below 0.001 $\mathrm{ppm}\;K^{-1}$ are displayed at the reporting floor.

The controlled benchmark maps the uniformly stretched material plane to an exact cylinder while curvature follows $\kappa \left(T\right)=0.04\left(T-25\right)/100\;{\mathrm{mm}}^{-1}$ for *T* in degrees Celsius. For the $0^{\circ }$ cylinder, the representative edge point at material coordinate s=10 mm has out-of-plane displacement $w=\kappa^{-1}\left[1-\cos\left(\kappa \lambda s\right)\right]$, which increases from 0 to 1.98 mm over 25–125 °C. Four curvature directions and three fixed ROIs give 72 curvature–direction–ROI conditions per estimator. The analytical mapping preserves the prescribed surface metric, so Figure 5 directly relates the imposed warpage history to estimator bias in this developable case.

Figure 5a shows the central $0^{\circ }$ sweep. At $\kappa =0.04\;{\mathrm{mm}}^{-1}$, global-coordinate DPF shifts by −68.88 $\mathrm{ppm}\;K^{-1}$ and local-plane DPF shifts by −7.04 $\mathrm{ppm}\;K^{-1}$. The surface-metric shift remains below the 0.001 $\mathrm{ppm}\;K^{-1}$ reporting resolution.

The same ranking persists when bending direction and ROI are changed (Figure 5b). At maximum curvature, the local-plane shift spans −6.31 to −8.87 $\mathrm{ppm}\;K^{-1}$ across the 12 direction–ROI combinations. Surface-metric shifts remain below the reporting resolution in all combinations. Table 2 summarizes the central $0^{\circ }$ results at maximum curvature. Over a 100 K excursion, the central maximum-curvature shifts correspond to approximately 6888 and 704 με for global and local DPF. The surface-metric shift is below 0.1 με at the selected display resolution.

### 3.4. Smooth Developable and Non-Developable Fields

The smooth S-shaped isometry contains a continuous, spatially varying curvature distribution. The surface metric recovers the prescribed material stretch. Projected-plane estimates respond to the local curvature sampled by their supports, extending the cylinder result to nonuniform developable warpage.

The Gaussian and saddle-like fields contain prescribed changes in the first fundamental form. Their surface-metric temperature slopes report the corresponding in-surface response of the reconstructed film or packaging layer. Projected-plane estimates additionally contain the geometric contribution created by planar parameterization.

### 3.5. Local-Operator Sensitivity

The fixed fifth-order, 9×9 operator was compared with cubic and quartic fits and with 7×7 and 11×11 supports. All configurations used the same coordinate sequences, ROI, and temperature regression. In the noise-free rows of Table 3, the reported value is the absolute CTE error; in the thermal-ramp noise rows it is the RMSE across 20 complete sequences. Cubic and quartic fits produced the same center-derivative stencil for the symmetric complete supports used here. At fixed 9×9 support, the fifth-order fit reduced deterministic curvature truncation from 0.00991 to $2.75\times 10^{-6}$ $\mathrm{ppm}\;K^{-1}$ for the cylinder and from 0.1575 to 0.0177 $\mathrm{ppm}\;K^{-1}$ for the smooth S-shaped field. Within the fifth-order family, the 9×9 support reduced noisy RMSE relative to 7×7 in all three geometries and retained smaller deterministic curved-field errors than 11×11. Based on this sensitivity test, the fifth-order, 9×9 operator was used for all subsequent analyses.

### 3.6. Coordinate-Noise Sensitivity of the Surface Metric

At the estimator input, stereo calibration, triangulation, and DIC correlation enter through their combined reconstructed-coordinate error. The noise study uses a 25×25 grid over $20\times 20\;{\mathrm{mm}}^{2}$, giving 0.833 mm grid spacing and a 6.67 mm span for the 9×9 operator. Six frames cover 25 to 125 °C at 20 K intervals. The three geometries are a flat film, a $30^{\circ }$ cylinder with $\kappa =0.04\;{\mathrm{mm}}^{-1}$, and the S-shaped isometry.

The isotropic input is $\alpha_{x}=\alpha_{y}=16.5$ $\mathrm{ppm}\;K^{-1}$, and the corresponding direct-Green–Lagrange regression reference is 16.5273 $\mathrm{ppm}\;K^{-1}$ in both directions. The reported scalar RMSE uses ${\hat{\alpha }}_{\mathrm{avg}}$. The reference field is exact. Independent zero-mean Gaussian noise is added to the five heated coordinate fields. In the constant high-noise condition, $\sigma_{X}=\sigma_{Y}=1\;\mu m$ and $\sigma_{Z}=5\;\mu m$. In the ramped condition, the heated-frame standard deviations are 0.36/0.36/1.8, 0.52/0.52/2.6, 0.68/0.68/3.4, 0.84/0.84/4.2, and 1/1/5μm in X/Y/Z. Each geometry–noise group contains 20 independent complete coordinate-field sequences.

**Figure 6:**
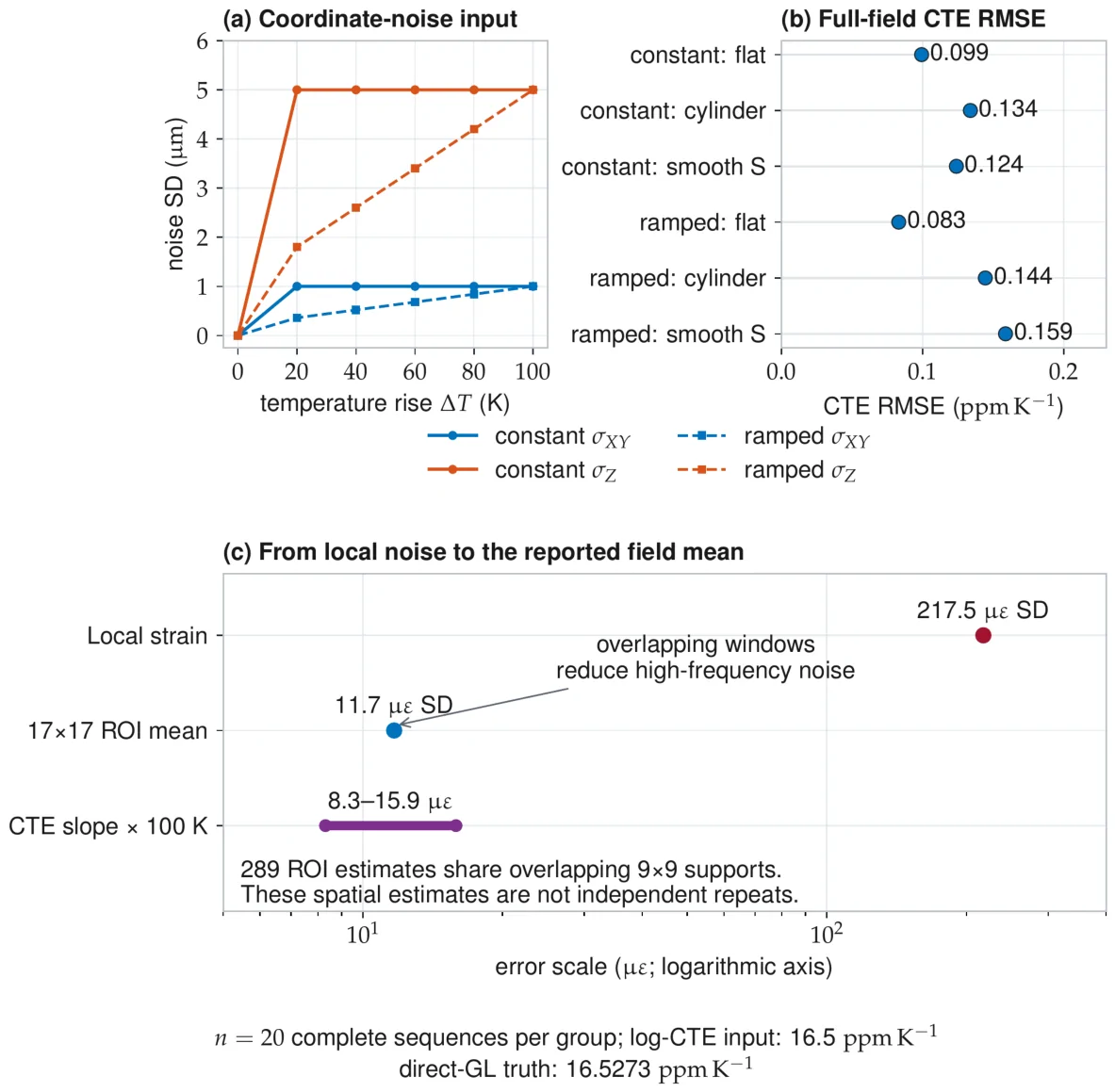
Coordinate-noise sensitivity. (**a**) Applied coordinate noise. (**b**) Surface-metric CTE RMSE. (**c**) Local-strain, ROI-mean, and sequence-level error scales.

Figure 6a states these noise inputs, and Figure 6b reports the six geometry–noise combinations separately. Across 20 complete sequences per group, the surface-metric CTE RMSE ranges from 0.0831 to 0.1587 $\mathrm{ppm}\;K^{-1}$.

In the flat constant high-noise condition, the predicted standard deviation of one local fifth-order mean-normal strain is 217.5 με. Propagating the derivative weights and overlapping supports through the 17×17 ROI gives a one-frame mean standard deviation of approximately 11.7 με. The complete six-frame sequences yield CTE RMSEs equivalent to 8.3–15.9 με over 100 K (Figure 6c). Field averaging reduces the one-frame strain uncertainty, and regression over the complete temperature sequence reduces its contribution to the CTE slope.

A separate regression sensitivity test perturbed the six temperature labels independently while holding the strain sequence fixed. For temperature standard deviations of 0.05, 0.10, 0.20, and 0.50 K, 1000 sequences gave CTE RMSEs of 0.0099, 0.0202, 0.0392, and 0.0982 $\mathrm{ppm}\;K^{-1}$, respectively; the absolute mean bias remained below 0.0021 $\mathrm{ppm}\;K^{-1}$. These values quantify the slope sensitivity for the stated temperature perturbations without changing the equal-frame regression.

## 4. Experimental Validation

### 4.1. Measurement Sequence and Analysis

Five specimens of supplier-specified 99.99% high-purity oxygen-free copper foil were tested. Each specimen measured $50\times 50\times 0.1\;{\mathrm{mm}}^{3}$ and rested on a glass plate. The plate provided mechanical support, and infrared radiation supplied the heat. Temperature was ramped at $1\;{}^{\circ}C\;s^{-1}$ and held for 30 s at each setpoint under the chamber’s integrated PID control. Each 50–290 °C experiment yielded interval CTE values for 24 consecutive 10 °C intervals.

The stereo system comprised two Hikrobot MV-CH250-90UM cameras (Hangzhou Hikrobot Technology Co., Ltd., Hangzhou, China) fitted with 75 mm lenses at a working distance of 1.2 m. Exposure was adjusted so that the specimen region remained below gray level 200 in the 8-bit images, leaving approximately 55 gray levels of headroom for heater glow. Stereo calibration used a 12×9 target with 10 mm spacing, Zhang’s calibration method, and iterative refinement of the target shape. The final reprojection error was 0.07 pixels. Stereo matching and reconstruction used in-house DIC software (V2.4.1) with a correlation core similar to OpenCorr. DIC used 51×51-pixel subsets with a 25-pixel step. The fixed experimental ROI comprised all valid DIC points in the observed specimen area. Acquisition and processing parameters are reported together following the measurement-reporting structure of the iDICs Good Practices Guide [38].

The analysis used the measured reference X,Y,Z coordinates and matched $D_{X},D_{Y},D_{Z}$ fields. Global-coordinate DPF, local-plane DPF, and the surface metric were evaluated on the same fixed ROI, with $E_{xx}$ and $E_{yy}$ averaged as separate directional fields before temperature regression. Global-coordinate DPF used a 7×7 support. Local-plane DPF and the surface metric used 9×9 supports.

The interval-wise reference CTE was calculated directly from the NIST Hahn1 copper thermal-expansion data and its published cubic-over-cubic rational fit [39,40]. The dataset contains 236 observations supplied by Thomas Hahn, with temperature expressed in kelvin. For each 10 °C interval, the fitted CTE was evaluated at the interval midpoint after adding 273.15 to the Celsius temperature. The full-precision fitted values were used to calculate deviations and summary statistics. The fit reports a residual standard deviation of 0.0818 $\mathrm{ppm}\;K^{-1}$, describing the scatter of the published observations around the fitted curve [40].

**Figure 7:**
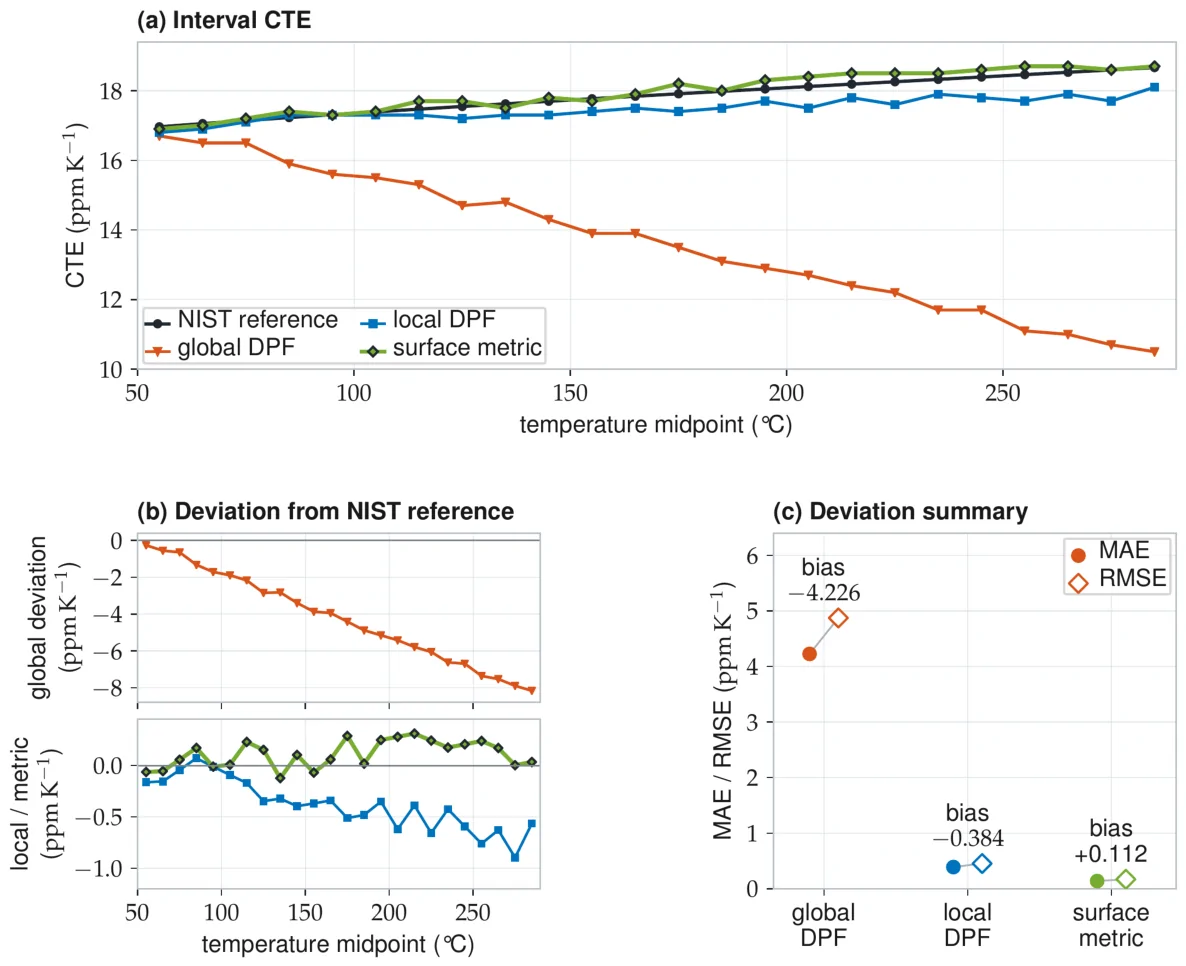
Copper-foil CTE results for one complete heating experiment. (**a**) Interval CTE. (**b**) Deviation from the NIST reference. (**c**) Deviation summary.

Figure 7 reports the 24 interval values and NIST-relative deviations for one complete experiment. Table 4 gives the interval values, and Table 5 summarizes their deviations.

**Figure 8:**
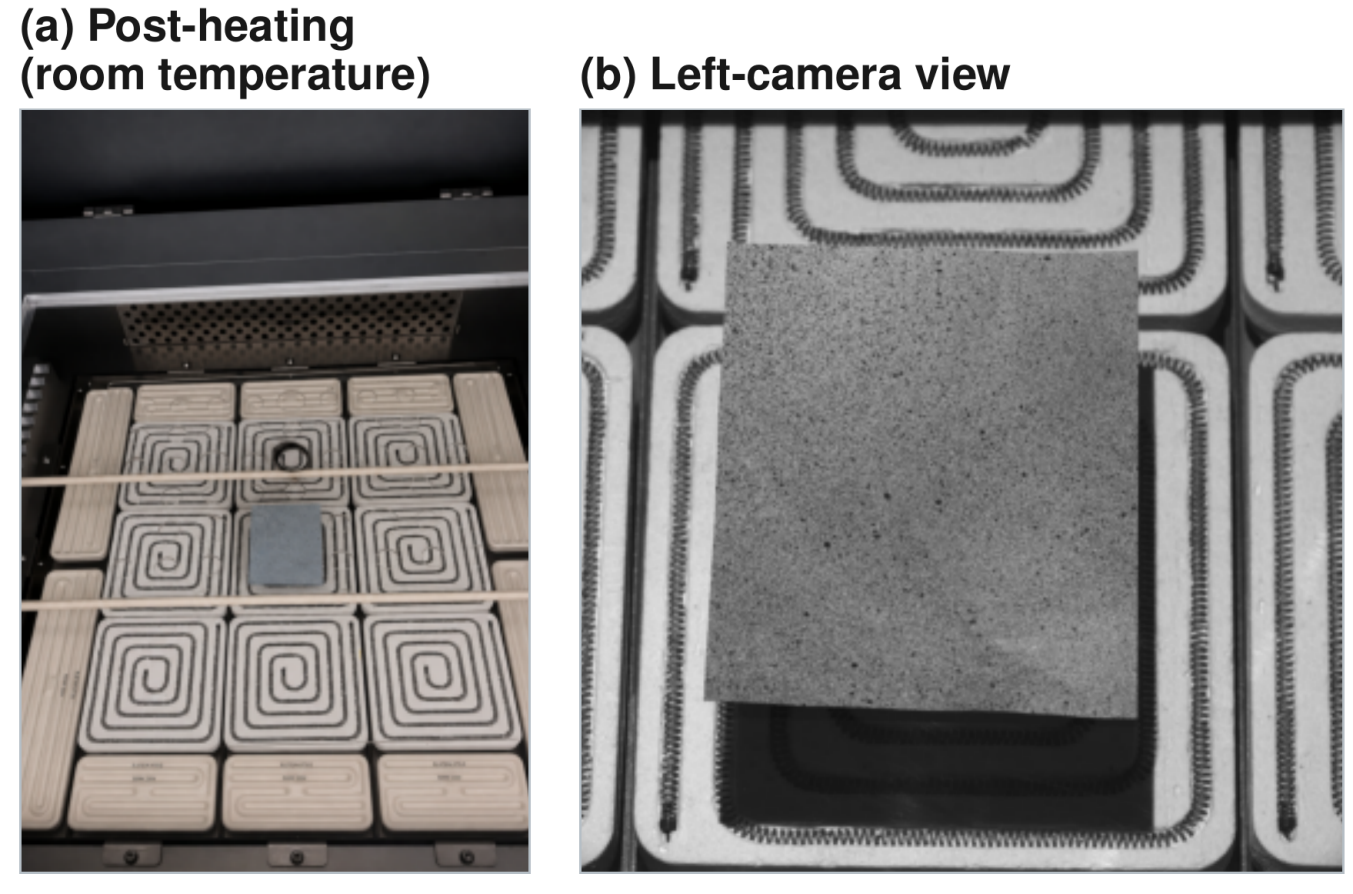
Copper-foil images of the 50×50 mm specimen. (**a**) Post-heating at room temperature. (**b**) Left-camera view. Both photographs are uncropped.

Figure 8 documents the specimen after heating at room temperature together with the left-camera view of the speckled copper foil.

### 4.2. Interval-Wise CTE Results

Table 4 gives the complete experimental series. The NIST reference CTE increased from 16.963 to 18.665 $\mathrm{ppm}\;K^{-1}$ over the interval midpoints. Global DPF moved in the opposite direction, decreasing from 16.7 to 10.5 $\mathrm{ppm}\;K^{-1}$. Its deviation was negative in all 24 intervals and widened from −0.263 to −8.165 $\mathrm{ppm}\;K^{-1}$. Local DPF followed the reference values more closely, but its deviation was predominantly negative and reached −0.897 $\mathrm{ppm}\;K^{-1}$ in the 270–280 °C interval. The surface-metric deviation ranged from −0.122 to +0.311 $\mathrm{ppm}\;K^{-1}$.

### 4.3. NIST-Relative Deviation Summary

With deviation defined as estimate minus the NIST reference CTE, global DPF had a mean signed deviation of −4.226 $\mathrm{ppm}\;K^{-1}$, MAE of 4.226 $\mathrm{ppm}\;K^{-1}$, and RMSE of 4.873 $\mathrm{ppm}\;K^{-1}$. Local DPF reduced these values to −0.384, 0.390, and 0.454 $\mathrm{ppm}\;K^{-1}$, respectively. The surface metric yielded a mean signed deviation of +0.112 $\mathrm{ppm}\;K^{-1}$, MAE of 0.139 $\mathrm{ppm}\;K^{-1}$, and RMSE of 0.170 $\mathrm{ppm}\;K^{-1}$. Its MAE was 64.4% lower than local DPF and 96.7% lower than global DPF. The corresponding RMSE reductions were 62.6% and 96.5%. The maximum absolute deviations were 8.165, 0.897, and 0.311 $\mathrm{ppm}\;K^{-1}$ for global DPF, local DPF, and the surface metric, respectively (Table 5).

The slope of signed deviation against interval midpoint provides an additional sequence-level measure. Global- and local-DPF slopes were −0.03501 and −0.003099, respectively, and the surface-metric slope was +0.000775. All slopes are expressed in $\mathrm{ppm}\;K^{-1}\;{{}^{\circ}C}^{-1}$. The surface-metric deviation trend was smaller than those of the two DPF estimates over the complete temperature range.

## 5. Discussion

### 5.1. Geometric Mechanism and Relation to Curved-Surface DIC

The cylinder sweep separates curvature-induced projection error from prescribed in-surface expansion. Global-coordinate DPF responds to laboratory-plane foreshortening, and local-plane DPF represents a finite curved patch by one average plane. Their CTE shifts grow with curvature and vary with bending direction and ROI. The first fundamental form uses the three-dimensional tangents themselves, so the tested developable bending changes surface orientation and shape while leaving the recovered material distances unchanged.

Tangent-frame, finite-element, isogeometric, and other surface-parameterized DIC formulations already provide curved-surface displacement and strain descriptions. The present contribution applies matched reference/current surface metrics to the specific metrology chain for film CTE: one material-coordinate derivative operator, reference-basis direct-Green–Lagrange components, a fixed ROI average, and a temperature slope. The controlled developable benchmark then supplies a direct test of curvature, direction, and ROI invariance against the two DPF implementations used for comparison.

The geometric formulation depends on matched surface coordinates and material-grid connectivity, without requiring elastic properties or a material-specific CTE as input. The cylinder sweep and smooth S-shaped isometry test its geometric invariance under uniform and spatially varying developable curvature. Copper foil provides the experimental evidence for the resulting CTE workflow. The Gaussian field evaluates a surface whose intrinsic metric changes, for which the estimator recovers the corresponding in-surface strain.

### 5.2. Coordinate Quality and Full-Field CTE Precision

Surface differentiation amplifies local coordinate fluctuations. The CTE estimate is calculated after component-wise field averaging and thermal regression. Under the tested 1μm in-plane and 5μm out-of-plane coordinate noise, the local-strain standard deviation was 217.5 με. The ROI-mean standard deviation was 11.7 με, and the complete-sequence CTE RMSE was 0.083–0.159 $\mathrm{ppm}\;K^{-1}$. This scale reduction explains why a noisy local-strain field can still yield a precise full-field CTE. Overlapping neighborhoods make adjacent strain points correlated, so the complete coordinate-field sequence is the independent unit in the Monte Carlo results.

This behavior also explains the need for field averaging in thermal DIC. A 1 $\mathrm{ppm}\;K^{-1}$ difference corresponds to only 1 με per kelvin. Under heated-air and optical disturbances, that increment is smaller than the uncertainty of one local differentiated estimate. Fixed-ROI averaging and temperature regression reduce the high-frequency random contribution. The surface-metric calculation removes the deterministic projection term retained by planar strain calculations.

A temperature-independent stereo scale factor multiplies the reference and current metrics equally and cancels from their ratio. Under the scale-drift model s(T)=1+βΔT, β is the corresponding additive contribution to directional CTE. The temperature-label simulation gives the complementary regression contribution: 0.10 K independent label noise produced 0.020 $\mathrm{ppm}\;K^{-1}$ CTE RMSE for the six-frame, 100 K sequence. Full-field averaging mainly attenuates high-frequency components. Experimental control of camera temperature, thermal shielding, and heat-haze distortion remains important for low-frequency affine drift.

### 5.3. Physical Interpretation and NIST-Referenced Comparison

The copper foils were evaluated under the support and heating conditions described in Section 4. Their directional slopes are compared with the published NIST copper reference using the same $E_{xx}$ and $E_{yy}$ workflow. The bias, MAE, and RMSE quantify agreement across the 24 intervals of the reported complete sequence. The surface metric follows both the magnitude and temperature trend of the reference more closely than the two DPF estimates.

## 6. Conclusions

In-plane CTE accuracy after 3D-DIC acquisition depends on the strain calculation. Global-coordinate DPF contains surface foreshortening, and independently fitted local planes contain a finite-patch projection term. Comparing matched first fundamental forms removes these projection contributions from smooth developable bending and yields separate $\alpha_{x}$ and $\alpha_{y}$ estimates.

The cylinder benchmarks recovered the prescribed direct-Green–Lagrange slopes across all bending directions and ROIs at the selected reporting resolution. Coordinate-noise simulations produced CTE RMSEs of 0.083–0.159 $\mathrm{ppm}\;K^{-1}$ after full-field averaging and temperature regression. In the reported complete copper-foil sequence, the surface metric yielded a mean signed deviation of +0.112 $\mathrm{ppm}\;K^{-1}$ and an RMSE of 0.170 $\mathrm{ppm}\;K^{-1}$ relative to the NIST copper reference.

The calculation uses the matched X,Y,Z and $D_{X},D_{Y},D_{Z}$ fields produced by 3D-DIC and does not require a prescribed warpage geometry. It retains the conventional fixed-ROI average and directional temperature regression used in CTE measurement.

## Figures and Tables

**Table 1:** Compared local-strain estimators.

Estimator	Local Geometric Object	Geometric Quantity Represented	Numerical Implication
Global-coordinate DPF	Affine fit to laboratory $D_{X},D_{Y}$ on the reference grid	Projected laboratory-plane length	Systematic dependence on global surface slope
Local-plane DPF	Affine displacement on one average window plane	Projected length on a fitted patch plane	Finite-window dependence on curvature
Surface metric	Smooth local tangents in matched material coordinates	Three-dimensional in-surface distance	Coordinate differentiation followed by component-wise full-field averaging

**Table 2:** Maximum-curvature CTE shifts relative to flat baselines.

Estimator	Identified Slope (ppm K^−1^)	Warpage-Induced Shift (ppm K^−1^)
Global-coordinate DPF	−48.841	−68.881
Local-plane DPF	13.002	−7.038
Surface metric	20.040	<0.001

**Table 3:** Local-operator sensitivity ($\mathrm{ppm}\;K^{-1}$).

Configuration	Cylinder Absolute Error	Smooth S Absolute Error	FlatRMSE	Cylinder RMSE	Smooth S RMSE
Cubic, 9×9	$9.91\times 10^{-3}$	0.1575	0.0777	0.0699	0.2021
Fifth order, 7×7	$5.13\times 10^{-7}$	0.00204	0.1419	0.1556	0.2108
Fifth order, 9×9	$2.75\times 10^{-6}$	0.0177	0.1100	0.1143	0.1706
Fifth order, 11×11	$9.94\times 10^{-6}$	0.0556	0.1146	0.1078	0.1439

Noise-free columns report absolute error; RMSE columns use thermal-ramp Gaussian coordinate noise and 20 complete sequences per geometry. All fits use the same central ROI and identical coordinate sequences.

**Table 4:** Copper-foil interval CTE values ($\mathrm{ppm}\;K^{-1}$).

Interval (°C)	NIST	Global	Local	Metric	Interval (°C)	NIST	Global	Local	Metric
50–60	16.963	16.7	16.8	16.9	170–180	17.911	13.5	17.4	18.2
60–70	17.055	16.5	16.9	17.0	180–190	17.981	13.1	17.5	18.0
70–80	17.143	16.5	17.1	17.2	190–200	18.051	12.9	17.7	18.3
80–90	17.228	15.9	17.3	17.4	200–210	18.120	12.7	17.5	18.4
90–100	17.311	15.6	17.3	17.3	210–220	18.189	12.4	17.8	18.5
100–110	17.392	15.5	17.3	17.4	220–230	18.257	12.2	17.6	18.5
110–120	17.470	15.3	17.3	17.7	230–240	18.325	11.7	17.9	18.5
120–130	17.547	14.7	17.2	17.7	240–250	18.393	11.7	17.8	18.6
130–140	17.622	14.8	17.3	17.5	250–260	18.461	11.1	17.7	18.7
140–150	17.696	14.3	17.3	17.8	260–270	18.529	11.0	17.9	18.7
150–160	17.769	13.9	17.4	17.7	270–280	18.597	10.7	17.7	18.6
160–170	17.840	13.9	17.5	17.9	280–290	18.665	10.5	18.1	18.7

NIST values are evaluated at interval midpoints and displayed to three decimal places; deviation statistics use the unrounded fitted values.

**Table 5:** NIST-relative deviations for the complete copper-foil sequence.

Method	Mean Signed Deviation (ppm K^−1^)	MAE (ppm K^−1^)	RMSE (ppm K^−1^)	Max. Abs. Deviation (ppm K^−1^)	Deviation-Trend Slope (ppm K^−1 ∘^C^−1^)
Global DPF	−4.226	4.226	4.873	8.165	−0.03501
Local DPF	−0.384	0.390	0.454	0.897	−0.003099
Surface metric	+0.112	0.139	0.170	0.311	+0.000775

Statistics describe the 24 intervals of one complete 50–290 °C sequence relative to the NIST fit. Global DPF and local DPF denote global-coordinate and local-plane DPF, respectively.

## Data Availability

The data and numerical strain-estimator source code supporting the findings of this study are available from the corresponding author upon reasonable request.
